# Lipoxin A4 attenuates the lung ischaemia reperfusion injury in rats after lung transplantation

**DOI:** 10.1080/07853890.2021.1949488

**Published:** 2021-07-14

**Authors:** Lijuan Zhang, Qihang Tai, Guangxiao Xu, Wei Gao

**Affiliations:** Department of Anesthesiology, The Second Affiliated Hospital of Harbin Medical University, Harbin, China

**Keywords:** Lipoxin A4, lung ischaemia reperfusion injury, lung transplantation

## Abstract

**Background:**

Lung ischaemia reperfusion injury (LIRI) is the major cause of primary lung dysfunction after lung transplantation. Lipoxin A4 inhibits the oxidative stress and inflammation. This study aimed to evaluate the potential protective effect of lipoxin A4 on LIRI in rats.

**Methods:**

SD (Sprague-Dawley) rats were randomised into the sham, LIRI and LA4 groups. Rats in the sham group received anaesthesia, thoracotomy and intravenous injection of saline, while those in the LIRI or LA4 group received left lung transplantation and intravenous injection of saline or lipoxin A4, respectively. After 24 h of reperfusion, the PaO_2_/FiO_2_ (Partial pressure of O2 to fraction inspiratory O2), wet/dry weight ratios and protein levels in lungs were measured to assess the alveolar capillary permeability. The oxidative stress response and inflammation were examined. The histological and apoptosis analyses of lung tissues were performed *via* HE staining (Haematoxylin-eosin staining) and TUNEL assay, respectively. The effects of lipoxin A4 on the endothelial viability and tube formation of hypoxaemia and reoxygenation-challenged rat pulmonary microvascular endothelium cells were determined.

**Results:**

Lipoxin A4 significantly ameliorated the alveolar capillary permeability, reduced the oxidative stress and inflammation in transplanted lungs. The histological injury and apoptosis of lung tissues were also alleviated by lipoxin A4. *In vitro* lipoxin A4 treatment promoted the endothelial tube formation and improved the endothelial viability.

**Conclusion:**

Lipoxin A4 protects LIRI after lung transplantation in rats, and its therapeutic effect is associated with the properties of anti-inflammation, anti-oxidation, and endothelium protection.

**Key messages:**
1. Lung transplantation is a treatment approach for the patients with lung disease.
2. LIRI is the major cause of postoperative primary lung dysfunction.
3. Lipoxins A4 exhibits strong anti-inflammatory properties.

## 1. Introduction

Lung transplantation is a major treatment approach for the patients with end-stage lung disease including cancer and pulmonary fibrosis. However, the lung ischaemia reperfusion injury (LIRI), which is characterised by alveolar damage, lung edoema and hypoxaemia, is the major cause of postoperative primary lung dysfunction [1,2]. In addition, LIRI is also the major risk factor for postoperative complications, such as acute graft rejection and obliterative bronchiolitis [3]. During the lung transplantation, the oxidative stress response, local inflammation, impairment of alveolar capillary permeability and cell apoptosis induced by LIRI significantly deteriorate the function and survival of transplanted lungs [4]. Although the significant advancement in clinical operation, graft preservation technologies, and pharmacological treatment, LIRI still occurs in about 20–35% patients [5], and had been identified to be the primary cause for the mortality of 14–18% patients in postoperative 90 days [6].

Lipoxins A4 is a product of arachidonic acid metabolism that exhibits strong anti-inflammatory properties [7]. It is characterised by inhibiting the neutrophil migration and accumulation [8], reducing the activities of p38 [9], PINK1 (PTEN induced putative kinase 1) signaling [10] and inhibiting NF-κB (Nuclear factor kappa-B) signalling [11] in multiple different models of organ injury. In addition, accumulating studies have demonstrated that lipoxin A4 can ameliorate various acute lung injury by balancing the inflammation and oxidative stress responses [10,12,13]. Lipoxin A4 has also been suggested to be able to mitigate the renal reperfusion injury [14]. However, the effect of lipoxin A4 on LIRI after lung transplantation has not been intensively studied so far.

According to the pathology of LIRI and the bioactive effect of lipoxin A4, we postulated that lipoxin A4 can ameliorate the LIRI *in vivo* and *in vitro*. In this study, using a lung transplantation model in rats, we investigated the impacts of lipoxin A4 administration on the alveolar capillary permeability, the histological injury, oxidative stress response, and apoptosis status in the grafted lung tissues. In addition, whether lipoxin A4 can affect the *in vitro* endothelial viability and tube formation of rat pulmonary microvascular endothelium cells was also investigated.

## 2. Materials and methods

### 2.1. Animals

Male Sprague Dawley rat (6 weeks old, total *n* = 34) were purchased from Charles River Laboratories (Beijing, China), and housed at the specific pathogen-free (SPF) facility at the Animal Centre of Harbin Medical University (Harbin, China) at room temperature (22 ± 1°C) with a 12/12 h light/dark cycle and access to food and water ad libitum. Rats were randomly assigned into the sham group, LIRI group and lipoxin A4 (LA4) group. Rats in the sham group only received the anaesthesia and thoracotomy, while rats in the LIRI and LA4 groups received the orthotopic rat left lung transplantation and injection of saline and lipoxin A4, respectively, as our previous studies [15,16]. This study was approved by the Secondary Affiliated Hospital of Harbin Medical University (SYDM2019-157). All treatments were carried out in accordance with the Institutional Animal Care and Use Committee of Second Affiliated Hospital of Harbin Medical University and followed national guidelines for the treatment of animals.

### 2.2. Donor graft preparation

A total of 18 donor rats were anaesthetised with intraperitoneal injection of 3% pentobarbital sodium (30 mg/kg body weight). After the disappearance of clip tail reflection, the rats were intubated and ventilated with the following parameters: tidal volume 10 ml/kg and 50 breaths per minute (50% O_2_ +50% N_2_) with 2 cm H_2_O positive end-expiratory pressure and inspiratory expiratory ratio of 1:1. Under analgesia with 1% lidocaine, rats were subjected to the thoracotomy. After heparinization with 300 U/kg heparin *via* femoral vein, the heart-lungs was collected, and followed by the flushing with 4 °C cold saline at 20 cm H_2_O pressure. Under the microscopy view, the left pulmonary artery, vein, and left bronchus were clipped on the CUFF tube (24 G central vein catheter). The graft was preserved at 4 °C for 60 min with end tidal volume.

### 2.3. Lung transplantation to recipients and intervention

A total of 16 recipient rats (8 rats in the LIRI group and 8 rats in the LA4 group) were anaesthetised as the donors and intubated with 12 G catheter. The femoral artery and vein were cannulated for blood collection and drug administration. After injection of 0.5 mg/kg atracurium, the recipients were ventilated with the same parameters as described for the donor rats. The anaesthesia was maintained with 1.5% sevoflurane. After left thoracotomy within 3 to 4 rib, the left lung was dislodged, and the pulmonary artery, vein and bronchus were dissociated. After blocking the blood and ventilation with clamps, the artery, vein and bronchus were anastomosed to CUFF tube of the lung graft. During this process, the tidal volume was decreased to 6 ml/kg and returned to 10 ml/kg after reperfusion. After closing the thoracotomy under local analgesia with 0.25% ropivaciane, the recipients were extubated, and followed with spontaneous breath. Rats in the sham and LIRI groups received intravenous injection of saline (1 ml each), while rats in the LA4 group received intravenous injection of lipoxin A4 (Cayman Chemical, Ann Arbour, MI, USA) at the dose of 100 μg/kg (diluted into 1 ml saline) after reperfusion, as previously described [12,14].

### 2.4. Lung samples collection

After 24 h of reperfusion, all the rats were anaesthetised with intraperitoneal injection of 3% pentobarbital sodium (30 mg/kg body weight) and cannulated. The arterial blood gas analysis was performed, and the peripheral blood was collected. After sacrificing the rats with overdose of anaesthetic, the left lungs were collected and divided into 3 parts. Upper part of the graft (the LIRI and LA4 groups) or control lung (the sham group) was stored at liquid nitrogen for further analysis of protein expression; the middle part was prepared for the histological and apoptotic evaluation; the lower part was homogenised with 0.9% saline (1: 9 weight) for testing the cytokines levels in the 10% homogenate. The peripheral blood and homogenate were centrifuged at 4 °C, 1000 g/min for 10 min, and the supernatant was collected for further analysis.

In this study, the observed time was referred previous studies, which suggested the the efficacy of LA4 can last for 24 h.

### 2.5. The alveolar capillary permeability

The partial pressure of O_2_ (PaO_2_) was analysed by the Bayer Rapidlab 348 Blood Gas Analyser (Bayer Diagnostics, Germany). The partial pressure of O_2_ to fraction inspiratory O_2_ (PaO_2_/FiO_2_) ratio was calculated. The lung tissues were weighed (wet weight) before drying for 72 h at 50 °C (dry weight). The wet/dry weight ratio was calculated by dividing the wet weight by the dry weight. The protein concentrations in the homogenate of lung samples were measured by the Bradford method [17].

### 2.6. Histological estimation

Part of lung samples was preserved in the paraffin, and the lung tissue was prepared for the HE staining to evaluate the histological injury, which was scored by 2 independent investigators. The score of lung injury was based on the Table 1 which included 5 variables such as lung haemorrhage, peri-bronchial infiltration of inflammatory cells, pulmonary interstitial edoema, pneumocyte hyperplasia and intra-alveolar infiltration of inflammatory cells. Each criterion was scored on a semiquantitative scale of 0–4, where 0 = normal, 1 = minimal change, 2 = mild change, 3 = moderate change and 4 = severe change. An overall histological score was calculated by summing the scores for criterion 1 through 5.

**Table 1.** Lung injury evaluation variables.

Parameters	Score
Haemorrhage	0 or 1
Peri-bronchial infiltration	0 or 1
Interstitial edoema	0 to 2
Pneumocyte hyperplasia	0 to 3
Intra-alveolar infiltration	0 to 3

### 2.7. Oxidative stress response

The activity of myeloperoxidase (MPO), superoxide dismutase (SOD), xanthine oxidase (XO), and the concentration of malondialdehyde (MDA) in the homogenate samples were measured with the specific kits (Nanjing Jiancheng, Nanjing, China) per the manufacturer’s instructions.

### 2.8. Inflammation assays

The systemic and local inflammation were assessed by testing the cytokines levels in homogenate and serum. The levels of tumour necrosis factor-α(TNF-α), Interleukin-1β (IL-1β) and Interleukin-10 (IL-10) in homogenate, as well as the levels of intercellular adhesion molecule-1 (ICAM-1) and monocyte chemotactic protein 1 (MCP-1) in serum, were determined by the Enzyme-Linked Immunosorbent Assays with commercial kits (Wuhan Boster Bio-Engineering Limited Company, Wuhan, China) following the manufacturer’s protocols. The activity of NF-κB in lung tissues was measured using the Transcription Factor Assay Kit (Abcam, Toronto, Canada) according to the manufacturer’s instructions. The expression of NF-κB in lung tissue was determined with Western Blot.

### 2.9. Western blot

Protein samples were prepared from the lung tissues using 1× cell lysis buffer (Cell Signalling Technology, USA). Equivalent amounts of proteins (10–20 µg) were used to assess protein expressions as described previously [11,12]. Briefly, protein was denatured by boiling, separated by sodium dodecyl sulfate-polyacrylamide gels (SDS-PAGE, 12%) and transferred to polyvinylidene difluoride (PVDF) membranes (Millipore, USA). The membranes were blocked with 8% non-fat milk/PBS-T (PBS with 0.5% Tween-20) for 2 h and respectively incubated with the following rabbit-derived primary antibodies: NF-κB, Bax, Bcl-xL, cleaved-Caspase-3 and β-actin (all 1:1000 dilution; Sigma Aldrich, St. Louis, MO, USA). After washing, the membranes were probed with horseradish peroxidase-conjugated goat anti-rabbit secondary antibody (1:5000 dilution; Cell Signalling Technology, USA). Proteins of interest were visualised using the enhanced chemiluminescence kit (EMD Millipore, USA), and the band intensities were quantified by densitometry using ImageJ software (National Institutes of Health, Bethesda, MD, USA).

### 2.10. Apoptosis assays

The lung tissue section for TUNEL (Terminal deoxynucleotidyl transferase dUTP nick end labeling) apoptosis assay was prepared according to the instruction of a commercial TUNEL kit (Roche Diagnostics GmbH, Science, Mannheim, Germany). Briefly, the lung tissue sections were treated with proteinase K and incubated in terminal deoxyribonucleotidyl transferase enzyme. And then, the sections were stained with diaminobenzidine–hydrogen peroxidase and Mayer’s haematoxylin. The nuclei with brown staining indicated apoptosis. Ten random fields of each section were selected, and two pathologists counted the apoptotic cells independently. The apoptosis index was calculated as the ratio of positive cells to total cells. In addition, the expressions of apoptosis associated proteins including Bax, Bcl-xL and cleaved-caspase-3 in lung tissues were evaluated by Western blot assays.

### 2.11. Endothelial viability and tube formation assays

Rat pulmonary microvascular endothelium cells were purchased from PriCells (Wuhan, China). The endothelium cells were cultured in normal endothelial cell growth medium supplemented with 10% foetal calf serum, 100 U/mL penicillin, 100 mg/mL streptomycin, and 1% endothelial cell growth factors under standard cell culture conditions (21% O_2_, 5% CO_2_, and 74% N_2_). To mimic hypoxaemia and reoxygenation, cells seeded in 96-well plates with a density of 1 × 10^4^ cells per well were cultured in glucose-deprived medium in a hypoxic chamber (5% CO_2_, and 95% N_2_; Biospherix hypoxia chamber, NY, USA) for 1 h, and then returned to the normal condition for 24 h. Subsequently, lipoxin A4 (100 ng/ml) was added in the culture medium of the LA4 group as previously described [18], and the cells in the LIRI group were treated with the vehicle PBS. Twenty-four hours after reoxygenation, all the cells were collected to test the viability and tube formation capacity.

The endothelial viability was evaluated using the cell counting kit-8 (CCK-8) commercial kit. Briefly, endothelium cells with a density of 1 × 10^4^ cells/well were plated in the 96-well plates. After incubation with the CCK-8 solution for additional 4 h, the absorbance of the culture medium was detected at 450 nm with a microplate reader (Quant Bio Tek Instruments, Winooski, Vermont, USA). The tube formation activity of endothelium was determined using the commercial assay kit (Abcam, Toronto, Canada) following the manufacture’s instructions. Briefly, 50 μl matrigel was added into the 96-well plate, and then the plate was incubated at 37 °C for 30 min. The endothelium cells (10^4^ cells/well) were seeded and cultured for 12 h. Then, the endothelium was washed with PBS and the tube network was imaged using the IX51 research microscope. Meanwhile, the tube formation was quantitatively measured using Image J software (National Institutes of Health, Bethesda, MD, USA).

### 2.12. Statistical analysis

All the data were presented as mean ± standard deviation (SD). All the variables were analysed by one-way analysis of variance. Data were analysed using the IBM SPSS Statistics 19.0 (SPSS, Chicago, IL, USA). Multigroup comparisons of the means were carried out by one-way analysis of variance (ANOVA) test with post hoc contrasts by Student–Newman–Keuls test. The statistical significance for all tests was set at *p* < 0.05.

## 3 Results

### 3.1. Lipoxin A4 ameliorated the alveolar capillary permeability in rats with lung transplantation

We established the *in vivo* model of lung transplantation in rats, as our previous reports [15,16]. To evaluate the effects of lipoxin A4 administration on lung ischaemia reperfusion injury, we treated the recipients with the vehicle or lipoxin A4 after transplantation, and compared multiple parameters including the alveolar capillary permeability, the histological injury, oxidative stress response, and apoptosis status in the grafted lung tissues.

**Figure 1.**
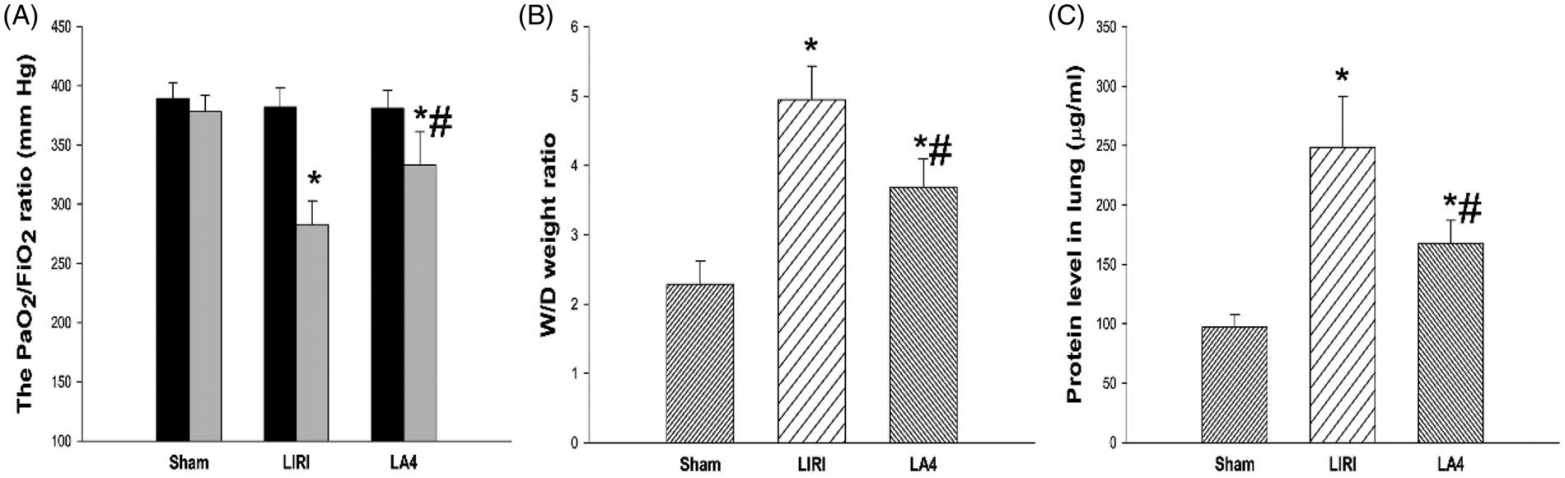
Lipoxin A4 ameliorated the alveolar capillary permeability after LIRI in rats. (A–C) Rats in the sham group only received the anaesthesia and thoracotomy, while rats in the LIRI and LA4 groups received the orthotopic rat left lung transplantation and injection of saline and lipoxin A4. The PaO_2_/FiO_2_ ratios before and at 24 hours after reperfusion in each group were calculated (A). The wet/dry weight ratio (B) and protein levels (C) in lung samples of rats in each group at 24 hours after reperfusion were determined. *n* = 8 rats for each group; *p <.05, *vs.* the sham group; #*p* <.05, *vs.* the LIRI group. 
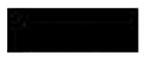
, baseline; 
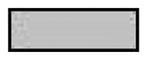
, 24 hours after reperfusion; 
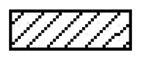
, the sham group; 
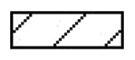
, the LIRI group; 
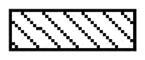
, the LA4 group.

As shown in Figure 1(A), while the control sham group demonstrated neglectable changes in the ratio of PaO_2_ to FiO_2_ after reoxygenation, the LIRI group and the LA4 group had significantly reduced PaO_2_ to FiO_2_ ratios after reperfusion. Compared with the LIRI group, the LA4 group displayed significantly improved PaO_2_ to FiO_2_ ratios. Similarly, after 24 h of reperfusion, compared with the sham group, the parameters including tissue wet/dry weight ratio (Figure 1(B)) and protein levels in lung tissues (Figure 1(C)) were worsened in the LIRI and LA4 groups. However, in comparison to the vehicle administration in the LIRI group, lipoxin A4 administration significantly mitigated the worsening of the wet/dry weight ratio (Figure 1(B)) and protein levels (Figure 1(C)).

### 3.2. Lipoxin A4 mitigated the histological injury of grafted lungs in rats

**Figure 2.**
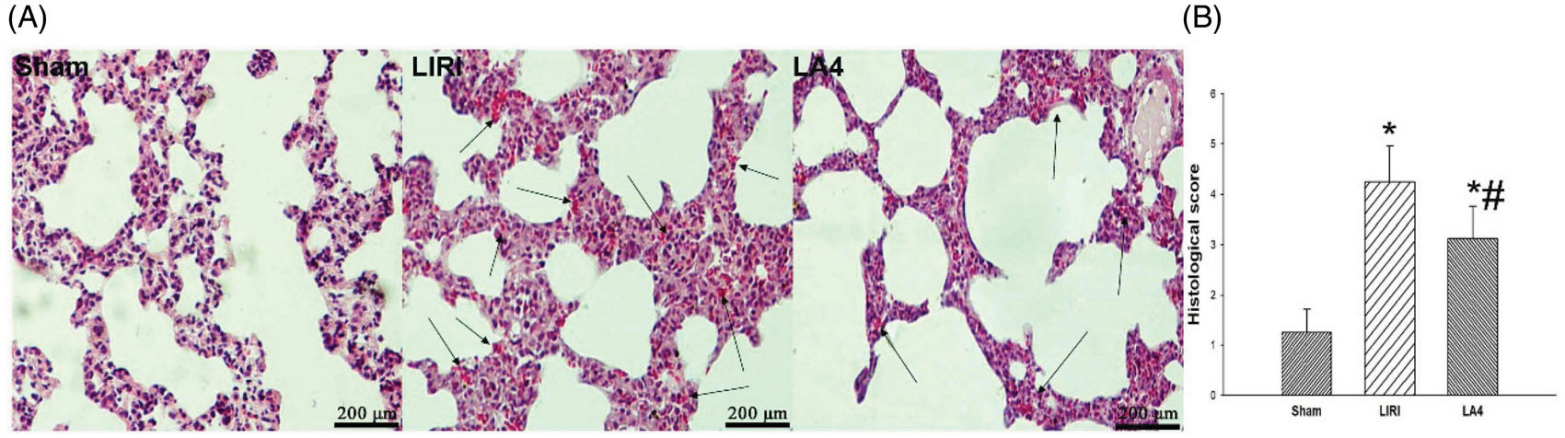
Lipoxin A4 attenuated the lung pathological injury in rats after lung transplantation. (A) Representative images of HE staining for the lung tissues in rats after 24 hours of reperfusion. Lots of inflammatory cells in the transplanted lung tissues, and severe thick alveolar wall and break alveolar wall were observed. There were severe edoema and haemorrhage in the alveolar tissues in the LIRI and LA4 groups. Scale bar, 200µm. (B) The score of histological injury was summarised. *n* = 8 rats for each group; *p <.05, *vs.* the sham group; #*p* <.05, *vs.* the LIRI group. 
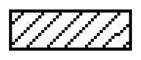
, the sham group; 
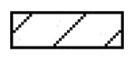
, the LIRI group; 
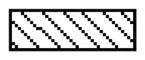
, the LA4 group.

At 24 h after reperfusion, the typical histological injury was observed in grafted lungs and control lungs. The histological changes of lungs included abnormalities in infiltration of inflammatory cells, severe alveolar and mesenchymal edoema, broken alveolar, and even the haemorrhage. Compared with the LIRI group, the treatment with lipoxin A4 significantly reduced the pathological injury, as evidenced by significantly decreased cellular infiltration, ameliorated lung edoema, and lessened alveolar injury (Figure 2(A)). Although the LA4 group still demonstrated significantly higher histological injury scores than the control sham group, lipoxin A4 was able to markedly lower these scores in the rats with lung transplantation (Figure 2(B)).

### 3.3. Lipoxin A4 suppressed the oxidative stress and inhibited inflammatory responses in grafted rat lungs

**Figure 3.**
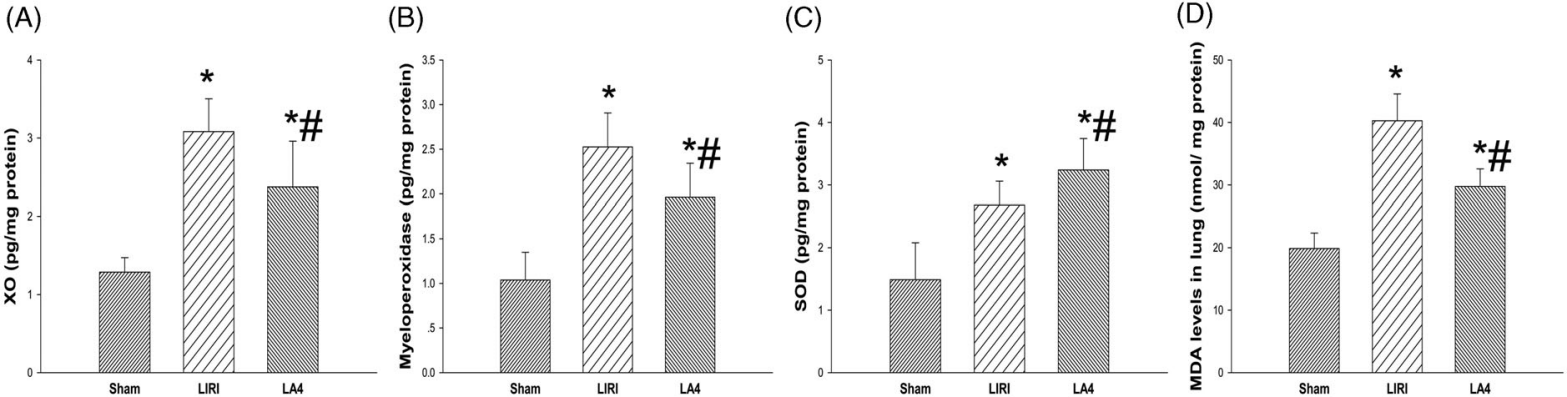
Lipoxin A4 inhibited the oxidative stress response in grafted lung tissues in rats. (A–D) The activity of XO (A), MPO (B) and SOD (C), and the concentration of MDA (D) in lung tissues of the indicated groups at 24 hours after reperfusion were measured. *n* = 8 rats for each group; *p < 0.05, *vs.* the sham group; #*p* < 0.05, *vs.* the LIRI group. 
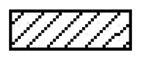
, the sham group; 
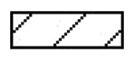
, the LIRI group; 
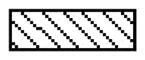
, the LA4 group.

We next investigated the impacts of lipoxin A4 on the oxidative and inflammatory responses of grafted lungs. The transplantation caused severe oxidative stress response in the lung tissues, as the lung tissues in the LIRI group and the LA4 group had significantly increased levels of XO (Figure 3(A)), MPO (Figure 3(B)), SOD (Figure 3(C)) and MDA (Figure 3(D)) in comparison to the lung tissues in the sham group. Compared with the LIRI group, the LA4 group demonstrated remarkably reduced levels of XO, MPO, and MDA. However, the treatment with lipoxin A4 rendered even higher levels of SOD in the grafted lungs (Figure 3).

**Figure 4.**
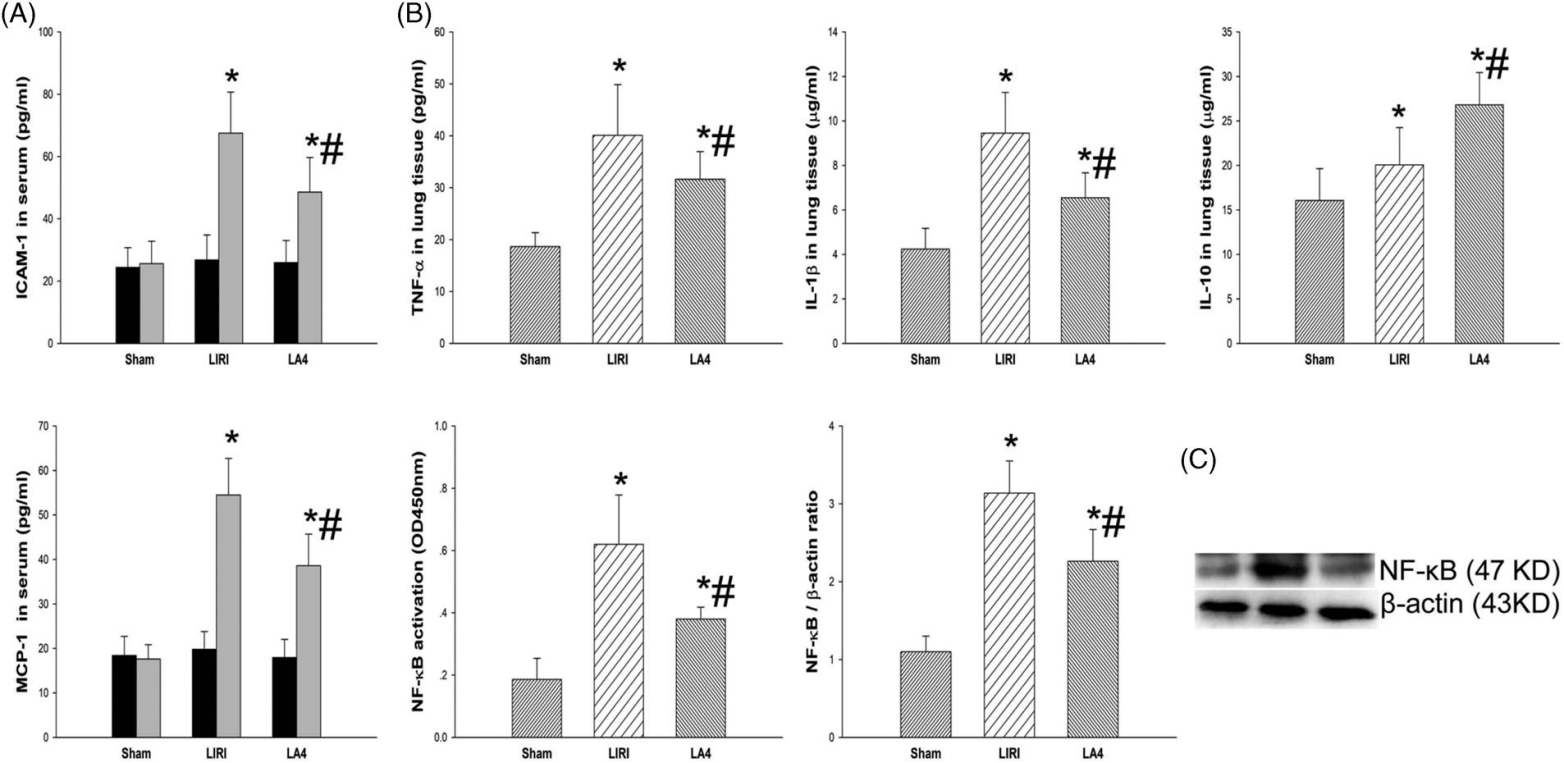
Lipoxin A4 attenuated the inflammation response associated with LIRI in rats. (A) The concentrations of ICAM-1 and MCP-1 in serum from the rats before and at 24 hours after reperfusion were measured. (B) The concentrations of TNF-α, IL-1β, and IL-10 in the control lung tissue and the transplanted lung tissue were measured. (C) The activity and expression of NF-κB in lung tissues at 24 hours after reperfusion were measured. Representative Western blot images are shown. *n* = 8 rats for each group; *p < 0.05, *vs.* the sham group; #*p* < 0.05, *vs.* the LIRI group. 
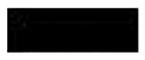
, baseline; 
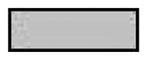
, 24 hours after reperfusion; 
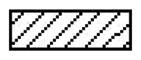
, the sham group; 
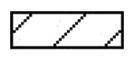
, the LIRI group; 
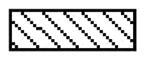
, the LA4 group.

The systemic and local inflammation was assessed by determining the cytokines levels in homogenate and serum samples, respectively. The lipoxin A4 treatment significantly down-regulated the levels of ICAM-1 and MCP-1 in the serum of rats after lung transplantation and reperfusion, although the lipoxin A4 treated rats still had significantly higher levels of ICAM-1 and MCP-1 than rats in the sham group (Figure 4(A)). In addition, after reperfusion, the levels of TNF-α, IL-1β and IL-10 in lung homogenate samples were significantly increased, especially for TNF-α and IL-1β, when compared with the sham group. Compared with the LIRI group, the LA4 group had significantly decreased levels of TNF-α and IL-1β, but increased levels of IL-10 (Figure 4(B)). Furthermore, the activity and expression levels of NF-κB in rat lung tissues were significantly up-regulated by the induction of ischaemia and reperfusion. Compared with the LIRI group, the activity and expression levels of NF-κB were significantly down-regulated by the treatment of lipoxin A4 in the LA4 group (Figure 4(C)).

### 3.4. Lipoxin A4 attenuated the apoptosis of lung tissues in rats after lung transplantation

**Figure 5.**
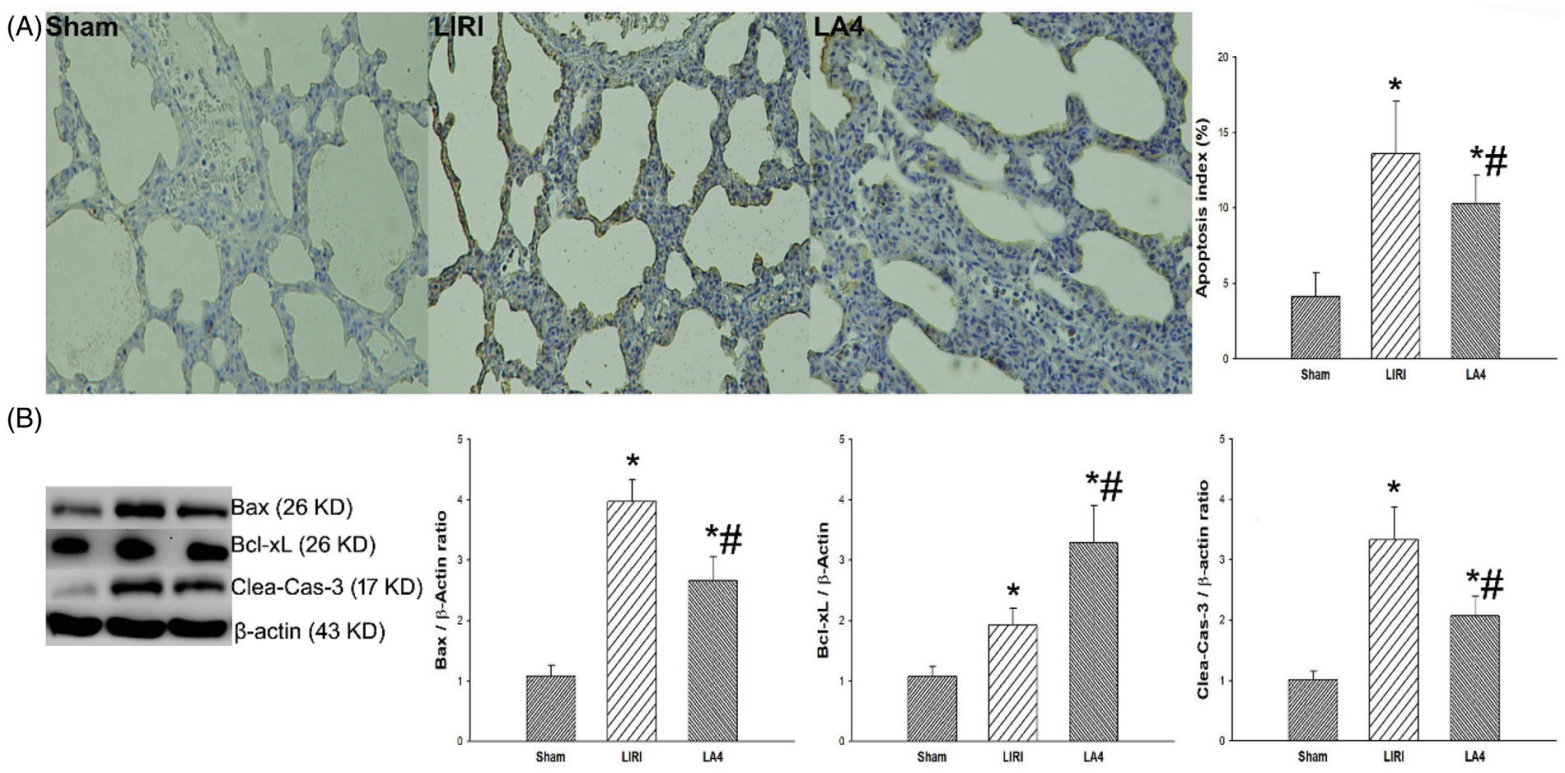
Lipoxin A4 inhibited the apoptosis of lung tissues induced by LIRI in rats. (A) Apoptosis of the lung tissues from the indicated groups at 24 hours after reperfusion was measured by TUNEL assays. Representative images are shown, and the relative apoptosis index was summarised. (B) The expression levels of Bax, Bcl-xL, cleaved-caspase-3 in the lung tissues of rats in the indicated groups were measured with Western blot. Representative images are shown, and the relative expression levels of the indicated proteins were summarised. β-actin was used as a loading control. *n* = 8 rats for each group; *p <.05, *vs.* the sham group; #*p* <.05, *vs.* the LIRI group. 
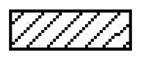
, the sham group; 
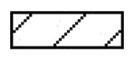
, the LIRI group; 
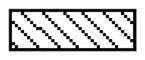
, the LA4 group.

There were lots of apoptotic cells in the transplanted lung tissue after reperfusion. We next examined how lipoxin A4 administration can affect the apoptosis of grafted lung tissues. Compared with the LIRI group, the LA4 group had significantly reduced number of apoptotic cells as determined by TUNEL assays (Figure 5(A)). After reperfusion, the expression levels of Bax, Bcl-xL and cleaved-caspase-3 proteins were significantly up-regulated in rats received lung transplantation in the LIRI and LA4 groups. Compared with the LIRI group, the LA4 group had significantly down-regulated expressions of Bax and cleaved-caspase-3 proteins, but up-regulated expression of Bcl-xL protein (Figure 5(B)).

### 3.5. Lipoxin A4 promoted the endothelial tube formation and improved the endothelial viability

**Figure 6.**
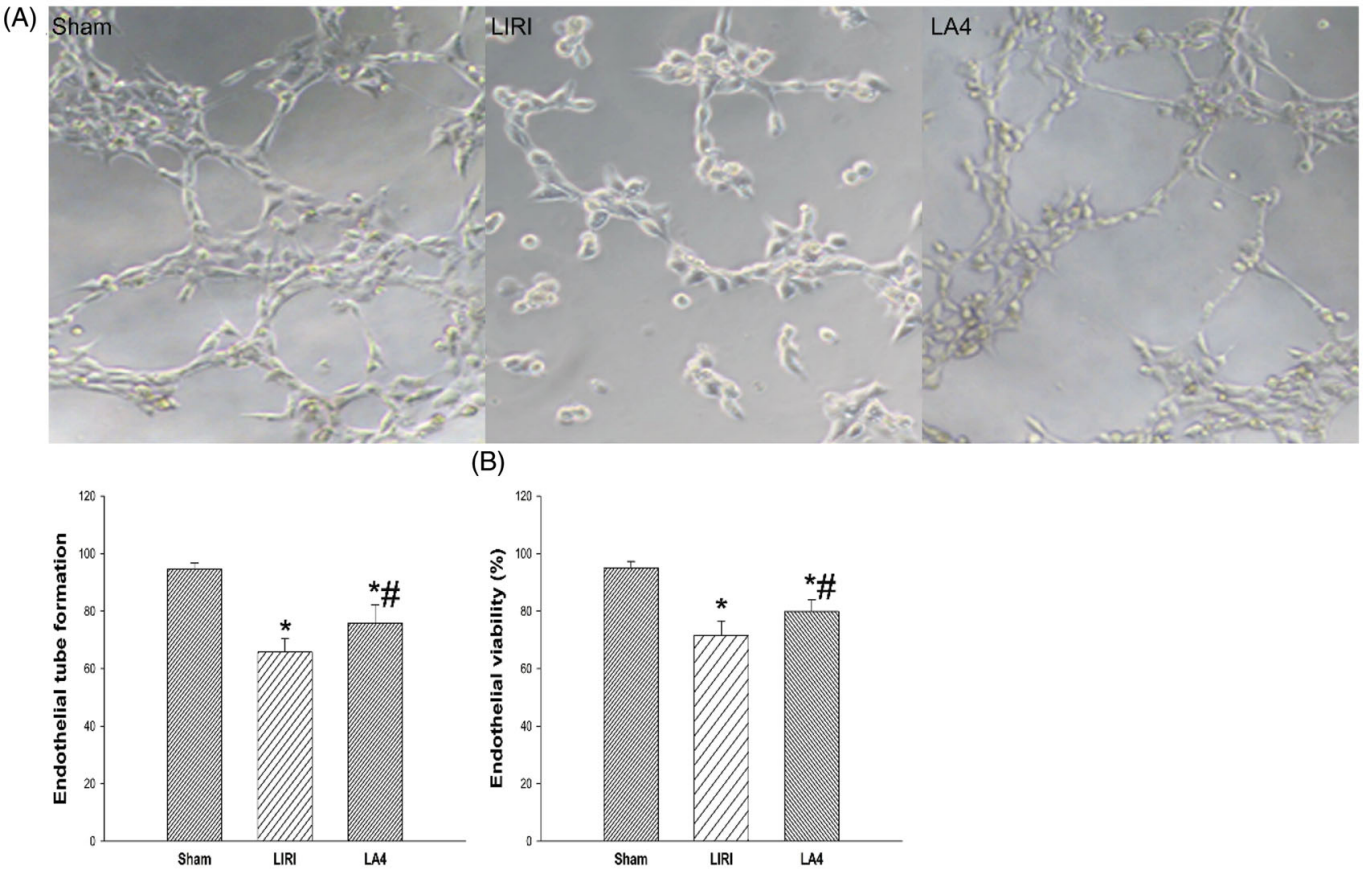
Lipoxin A4 promoted the endothelial tube formation and improved the endothelial viability. (A,B) After reoxygenation, the *in vitro* tube formation capacity (A) and the viability (B) of rat pulmonary microvascular endothelium cells in the indicated groups were determined. Representative images of endothelium tube formation are shown, and the relative tube formation capacity was summarised. However, the lipoxin A4 significantly improved the endothelial viability and tube formation capacity. *n* = 3 for each group; *p < 0.05, *vs.* the sham group; #*p* <.05, *vs.* the LIRI group. 
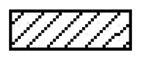
, the sham group; 
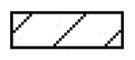
, the LIRI group; 
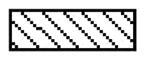
, the LA4 group.

It had been indicated that lipoxin A4 exhibited the anti-inflammation property mainly due to the direct effect on the inflammation status of the endothelium cells in cardiovascular disease [19]. To investigate the effects of lipoxin A4 on the endothelium function, we established an *in vitro* cellular hypoxaemia and reoxygenation model to study whether lipoxin A4 administration could directly protect the endothelium. After reoxygenation, the tube formation of the endothelium cells was deteriorated in the LIRI and LA4 groups. Compared with the LIRI group, the LA4 group had significantly enhanced capacity of endothelial tube formation (Figure 6(A)). Moreover, the viability of endothelium after reoxygenation was significantly decreased in the LIRI and LA4 groups in comparison to the sham group. However, the decrease of endothelial viability was partially reversed by the lipoxin A4 administration (Figure 6(B)).

## 4. Discussion

As a common and severe complication after lung transplantation, the LIRI is still the major cause of death during early period after lung transplantation [1,2]. Although the pathology of LIRI is complex and still not fully elucidated, inflammation, oxidative stress response, and endothelium dysfunction have been indicated to play a pivotal role [3]. In this study, we found that lipoxin A4 significantly ameliorated the lung injury after lung transplantation in rats. Lipoxin A4 not only reduced the ischaemia reperfusion induced inflammation, oxidative stress response and apoptosis in transplanted lung tissues, but also improved the endothelial function *in vitro*.

Firstly, we performed assays on capillary permeability and histological injury to estimate the effect of lipoxin A4 on LIRI in rats with lung transplantation. We found that lipoxin A4 significantly reduced the lung histological injury, upregulated the PaO_2_/FiO_2_ ratio, and down-regulated the wet/dry weight ratio and protein levels in lung tissue. These indicators suggest that the lipoxin A4 ameliorated the LIRI after lung transplantation. Considering the important role of oxidation in LIRI, we estimated the effect of lipoxin A4 on oxidative stress response. During hypoxaemia and reoxygenation, the anaerobic metabolism produces lots of hypoxanthine, which will be degraded to generate the reactive oxygen species under the activation of XO [20]. XO induced superoxide formation and accumulation plays a major role in oxidative stress during IR injury. Moreover, the neutrophils play an important role in oxidative mechanisms in LIRI *via* acting on the MPO system. In activated neutrophils, MPO produces the hypochlorite salts through combined H2O2 generation and release of the oxygen free radical. MPO and MPO derived oxidants are important factors in the pathogenesis of organ ischaemia reperfusion injury [21,22].

In contrast to XO and MPO, SOD is an important anti-oxidative enzyme and can be activated to eliminate superoxide anion. As the final product of lipid peroxidation, MDA is the final product of oxidative stress, and its expression level represents the severity of oxidative stress response. In this study, we found that lipoxin A4 significantly reduced the concentration of MDA in grafted lung tissue. This result suggests that lipoxin A4 can inhibit the oxidative stress after LIRI. The anti-oxidative effect of lipoxin A4 may be due to its regulation on the levels of XO, MPO and SOD. These results indicate that lipoxin A4 down-regulated the activity of XO and MPO, but increased the activity of SOD, which contributed to the suppression of oxidative stress response induced by the lung reperfusion injury. This result is in agreement with the conclusion of previous studies [14,23].

Secondly, we evaluated the effect of lipoxin A4 on inflammation of grafted lung tissues in rats after LIRI. During ischaemia and reperfusion, the hypoxaemia and reoxygenation could lead to endothelial injury, and the NK-κB signalling of damaged endothelium can be activated, which renders the release of the chemoattractant such as ICAM-1 and MCP-1 into serum [24,25]. Under activation by the chemoattractant, the inflammatory cells are recruited into the transplanted lung and then secret lots of cytokines. In this study, we found that lipoxin A4 significantly decreased the release of chemoattractant ICAM-1 and MCP-1, and thus inhibited the chemoattraction of inflammatory cells, and further decreased the secretion of TNF-α and IL-1β. The suppressive role of lipoxin A4 on inflammation mainly depended on the inhibition of NF-κB signalling. NF-κB is a key regulator of inflammation [26], and the inhibition of NF-κB can alleviate the LIRI [27]. During LIRI, NF-kB translocates into the nucleus and activates the pro-inflammatory factor genes, and thus promotes the release of inflammatory factors including TNF-α and IL-1β, which finally results in organ injury [27]. In this study, we found that lipoxin A4 significantly down-regulated the expression of NF-κB and lessened the activity of NF-κB, which was consistent with previous study [11]. Furthermore, the role of lipoxin A4 in regulating inflammation was also associated with the promoted production of IL-10 [12], which is an important anti-inflammatory factor that can weaken the LIRI [28].

Either reactive oxygen species or proinflammatory factors produced by LIRI can lead to apoptosis of transplanted lung tissues, which directly determined the function of transplanted lung [29,30]. In this study, lipoxin A4 significantly reduced the degree of apoptosis in the transplanted lung tissues. To investigate the possible mechanism underlying the anti-apoptotic role of lipoxin A4, we evaluated the effect of lipoxin A4 on apoptosis-associated proteins. We found that lipoxin A4 significantly down-regulated the expressions of Bax and cleaved-caspase-3, but up-regulated the expression of Bcl-xL. Bax is an important pro-apoptotic protein, which can initiate the intrinsic apoptosis pathway [31], and further activate the cleavage of caspased-3. Contrast to Bax, Bcl-xL is a pivotal anti-apoptotic protein, which can block the intrinsic apoptosis by inhibiting the release of Bax [32] or extrinsic apoptosis by inhibiting the activation of Bid [33]. Our results implied that the antiapoptotic effect of lipoxin A4 was associated with the regulation on the expressions of Bax, Bcl-xL and cleaved-caspase-3.

The endothelial injury induced by oxidative stress and inflammation is the key factor in LIRI [34]. To further explore the protective role of lipoxin A4 on lung injury after lung transplantation, we examined the effect of lipoxin A4 on endothelial viability and function. In the *in vitro* cellular experiment, we found the viability and the tube formation capacity of endothelium were significantly deteriorated by hypoxaemia and reoxygenation. However, the treatment with lipoxin A4 significantly weakened the worsening of endothelial viability and tube formation capacity. These results suggested that the protective role of lipoxin A4 in LIRI not only depended on the inhibition of oxidative stress, inflammation, and apoptosis, but was also associated with the protection on endothelial viability and function.

## 5. Conclusion

Lipoxin A4 can ameliorate the LIRI in rats after lung transplantation, which is associated with the ability of lipoxin A4 in suppressing the oxidative stress, inflammation, and apoptosis of lung tissues, as well as its protection on the endothelial viability and tube formation capacity. Our study sheds new light on the clinical application of lipoxin A4 in the patients with lung transplantation.

## Data Availability

The datasets used and analysed during the current study are available from the corresponding author on reasonable request.

## Abbreviations

ICAM-1: intercellular adhesion molecule-1
LA4: lipoxin A4
LIRI: lung ischaemia reperfusion injury
MCP-1: monocyte chemotactic protein 1
MDA: malondialdehyde
MPO: myeloperoxidase
PaO_2_: partial pressure of O_2_
SOD: superoxide dismutase
TNF: tumour necrosis factor
XO: xanthine oxidase.
